# Case of Acute Disseminated Encephalomyelitis Associated with Cytomegalovirus Reactivation in an Immunocompromised Systemic Lupus Erythematosus Patient

**DOI:** 10.3390/medicina57090882

**Published:** 2021-08-27

**Authors:** Mirela Pavicic Ivelja, Kresimir Dolic, Daniela Marasovic Krstulovic, Gordana Glavina, Ivo Ivic

**Affiliations:** 1 Department of Infectious Diseases, University Hospital of Split, University of Split School of Medicine, Soltanska 1, 21 000 Split, Croatia; iivic@kbsplit.hr; 2 Department of Radiology, University Hospital of Split, University of Split School of Medicine, Spinciceva 1, 21 000 Split, Croatia; kdolic79@gmail.com (K.D.); gordana.glavina@st.net.hr (G.G.); 3 Department of Rheumatology and Clinical Immunology, University Hospital of Split, University of Split School of Medicine, Soltanska 1, 21 000 Split, Croatia; daniela.marakrst@gmail.com

**Keywords:** cytomegalovirus, encephalomyelitis, demyelinating diseases, lupus erythematosus, systemic

## Abstract

We present a case of an immunocompromised systemic lupus erythematosus female patient admitted to our hospital for general impairment, monoparesis, and temporary cognitive disability. The case represented a significant diagnostic and therapeutic challenge primarily because of a wide range of differential diagnosis options (CNS lupus, ischemic cerebrovascular disease, viral meningoencephalitis, progressive multifocal leukoencephalopathy, limbic encephalitis, and acute disseminated encephalomyelitis—ADEM). Brain MRI findings were compatible with ADEM, and microbiological tests showed a cytomegalovirus infection (CMV) which is rarely associated with ADEM despite the increasing number of immunocompromised patients prone to symptomatic CMV reactivation. Our patient was treated with intravenous methylprednisolone, immunoglobulin (IVIG), along with antiviral therapy resulting in a favorable therapeutic effect. In conclusion, only a few described ADEM cases have been associated with CMV, and none of them, to the best of our knowledge, in an immunocompromised patient. In this case, a multidisciplinary approach and broad diagnostic considerations were decisive for successful treatment and outcome.

## 1. Introduction

The spectrum of human illnesses caused by cytomegalovirus (CMV) is diverse and mostly dependent on the host. CMV is often acquired early in life. In immunocompetent individuals, primary CMV infection rarely causes an illness typically running a benign, self-limited course. Like other members of the Herpesvirus family, CMV establishes a latent infection after the resolution of the primary infection. The reactivation of the latent virus causes a majority of the symptoms while hosts are in an immunocompromised status resulting in substantial morbidity and mortality.

Acute disseminated encephalomyelitis (ADEM), also known as post-infectious encephalomyelitis, is a rare autoimmune demyelinating disease of the central nervous system triggered by an environmental stimulus in genetically susceptible individuals. ADEM is caused by an inflammatory reaction in the brain and the spinal cord, and its onset is acute and often rapidly progressive occurring more frequently in children than adults. It is typically monophasic, but some patients may either have recurrences or have an ADEM-like presentation as the first attack of a chronic demyelinating disease such as multiple sclerosis (MS) or neuromyelitis optica [1]. ADEM is associated with a preceding infection or vaccination in 50 to 75 percent of cases, but among cases with an identified precipitant, most are associated with natural infection and only a small minority with vaccination. Although frequently preceded by a viral or bacterial infection, an underlying pathogen is often not identified and ADEM may follow a nonspecific upper respiratory or gastrointestinal illness. The list of numerous infectious pathogens associated with ADEM includes rubella, mumps, varicella (VZV), measles, smallpox, Epstein-Barr virus, herpes simplex virus (HSV), human herpes virus-6, influenza, human immunodeficiency virus, Mycoplasma pneumoniae, etc. Only a few described cases have been associated with CMV and none of them, to the best of our knowledge, in an immunocompromised patient. ADEM associated with systemic lupus erythematosus (SLE) has rarely been reported [2,3,4,5].

We report a case of ADEM secondary to the CMV infection in a patient with SLE that improved with intravenous methylprednisolone and immunoglobulin (IVIG) along with an antiviral therapy.

## 2. Case Presentation

**Figure 1:**
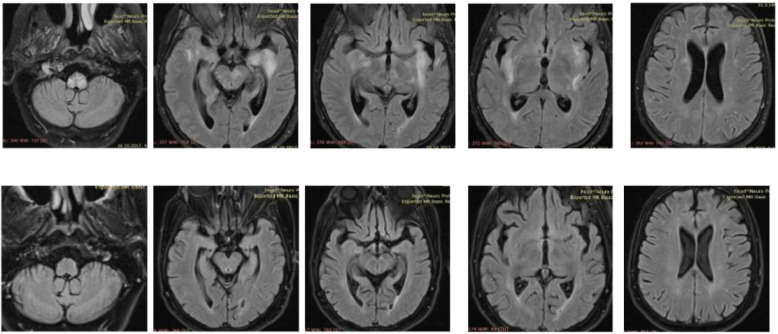
Brain MRI: multiple parenchymal supratentorial and brainstem demyelinating lesions on T2 and FLAIR sequences without postcontrast enhancement on T1WI (upper row); follow-up MRI six months after hospitalization showing complete regression (bottom row).

A 57-year old immunocompromised Caucasian woman with a diagnosis of SLE in stable remission for the last nine years and treated with low dose prednisone was admitted to the Internal Medicine Ward because of a poor general condition lasting for seven days and weakness in the right leg without sensory deficit lasting for two days. During that time, she became disorientated and confused, while meningeal signs were negative. Laboratory tests showed a sedimentation rate of 26 mm/h, leucocytes 3.9 × 10^9^/L with neutrophilia, erythrocytes 4.38 × 10^12^/L with thrombocytopenia of 125 × 10^9^/L, mild liver lesion with aspartate aminotransferase (AST) 43 U/L, and alanine aminotransferase (ALT) 71 U/L. C3 and C4 complement components were normal, as well as immunoglobulins level (IgA 0.91 g/L, IgG 10.1 g/L, and IgM 1.67 g/L). Antiphospholipid antibodies were positive (aCL-IgM 69.1 MPL/mL, aCL-IgG 88.8 GPL/mL), ANA titar (IIF) was 1:1280, and dsDNA (ELISA) 52 IU/mL (normal < 30 IU/mL). An urgent brain MSCT was performed and showed discrete hypodense lesions of the left perinsular and hippocampal region. Due to the previous medical history, clinical presentation and initial imaging findings, central nervous system (CNS) lupus, and subacute ischemic cerebrovascular disease were suspected. Follow-up CT after 5 days showed a new lacunar hypodense lesion in the left cerebellar peduncle. Next, MR angiography was performed excluding signs of vascular anomalies, ischemia, and vasculitis. In the second week, the patient showed further deterioration of clinical symptoms with a decrease in the leucocyte count (L 2.7 × 10^9^/L), normocytic anaemia (hemoglobin 109 g/L), and thrombocytopenia (platelets 92 × 10^9^/L). C3 was 0.83 g/L and C4 was normal. Brain MRI in the second week of hospitalization revealed the full extent of brain impairment: multiple parenchymal supratentorial and brainstem demyelinating lesions on T2 and FLAIR sequences without postcontrast enhancement on T1WI (Figure 1).

Since we had an immunocompromised patient, CMV serology (ELISA) was also carried out with both IgM and IgG resulting in positive as well as blood CMV polymerase chain reaction (PCR) that revealed 4340 CMV copies per milliliter. A lumbar puncture was performed. Cerebrospinal fluid (CSF) analysis showed elevated protein at 1567 mg/L with normal glucose and lactate, 6 leucocytes/mm^3^, 645/mm^3^ erythrocytes, and negative bacterial culture. PCR detected CMV DNA in the CSF (<1000 copies/mL), while PCR for HSV and VZV were negative. There was no intrathecal oligoclonal bands synthesis. Clinical findings, course of the disease, laboratory tests (lower leucocytes, thrombocytopenia, hepatal lesion), neuroradiological features along with serology, and PCR tests suggested an ADEM associated with CMV reactivation. Antiviral therapy with gancyclovir and subsequently with peroral valganciclovir was administered for 29 days in total (until CMV PCR came negative in CSF and blood). High doses of intravenous methylprednisolone (125 mg intravenously for five days with consequent gradual dose reduction) and intravenous immunoglobulin (IVIG) 0.4 g/kg daily for three days were started simultaneously.

The patient‘s neurological status gradually improved. At the discharge, almost a month after admission, the patient was completely conscious with improved right leg paresis. Follow-up brain MRI, three weeks after the initial one, revealed partial regression of previously described demyelinating lesions. Further, a follow-up clinical examination 6 months after hospitalization showed mild right leg paresis and complete MRI regression of demyelinating lesions (Figure 1) Our patient had no similar recurrent attacks since.

## 3. Discussion and Conclusions

ADEM is an immune-mediated inflammatory and demyelinating disorder of the CNS often triggered by bacterial and viral infections. CMV is a recognized cause of morbidity and mortality in immunocompromised individuals but, until now, it has not been linked to ADEM in this constantly growing worldwide population [6,7].

Our patient represented a significant diagnostic and therapeutic challenge primarily because of a wide range of differential diagnosis options. At first, before the MRI was performed, CNS lupus was suspected. Leucopenia, anemia, thrombocytopenia, and C3 complement slightly lower than normal range with mild positivity of dsDNA were indicating lupus flare with CNS involvement. MRI findings, later on, diverted us from that diagnosis because of untypical lesions distribution with no signs of infarctions or stenotic arterial lesions commonly found on an MR or MR angiography. In addition, the above-mentioned laboratory abnormalities including leucopenia, thrombocytopenia, and elevated liver enzymes can be explained by CMV reactivation provoking post or, in this case, a rather para-infectious immune-mediated demyelination. Even though it is not easy to reveal a causal relationship between CMV infection and ADEM, we think that in our case it was a probable trigger for demyelinating disease development.

Subacute ischemic cerebrovascular disease was also suspected but follow-up CT and MR angiography excluded ischemia. Progressive multifocal leukoencephalopathy (PML) as a demyelinating inflammatory disease of the brain was included in the differential diagnosis, but CSF PCR for the JC virus was negative. Although CSF PCR for CMV was positive, it was clear that this was not just a case of simple CMV encephalitis, especially with demyelinating lesions on brain MRI and having a favorable therapeutic response with methylprednisolone and IVIG.

Differential diagnosis also included limbic encephalitis but the distribution of the lesions on the MRI did not match: the usual location of involvement are the mesial temporal lobes and limbic systems, typically manifested by cortical thickening and increased T2/FLAIR signal intensity of these regions [8]. Based on the MRI findings, MS-like brain changes, sometimes termed “lupoid sclerosis”, could have been considered but the distribution of the lesions was not characteristic and there was no intrathecal oligoclonal bands synthesis.

In conclusion, although CMV is only extraordinarily associated with ADEM, it must be included in microbiological tests, especially in immunocompromised patients, since they are prone to this infection and early diagnosis and treatment ameliorate the neurological outcome. Broad differential diagnosis was the major challenge with our patient’s clinical and imaging presentation. Once more, as often proved with immunocompromised patients, a multidisciplinary approach was crucial in diagnosis and treatment decisions resulting in a favorable outcome.

## Data Availability

The data presented in this case are available on request from the corresponding author.
